# The distribution of lateral rib fractures: a validation and further development of the AO/OTA classification system in patients with fractures at the rib shaft

**DOI:** 10.1007/s00068-025-02795-w

**Published:** 2025-02-23

**Authors:** Johannes Groh, Florian Kern, Johannes Krause, Mario Perl, Stefan Schulz-Drost

**Affiliations:** 1 Department of Trauma and Orthopedic Surgery, University Hospital Erlangen, Friedrich-Alexander University Erlangen-Nürnberg (FAU), Krankenhausstraße 12, 91054 Erlangen, Germany; 2 Faculty of Medicine, Department of Anesthesiology, Friedrich-Alexander-Universität Erlangen-Nürnberg, Erlangen, Germany; 3 Department for Trauma Surgery, Helios Kliniken Schwerin, Schwerin, Germany

**Keywords:** Rib fracture, Flail chest, Chest wall injury, Rib shaft, AO/OTA classification system

## Abstract

**Introduction:**

Rib fractures are prevalent and clinically significant injuries, often associated with thoracic trauma. Despite their frequency, the precise distribution and characteristics of rib shaft fractures remain underexplored. This study investigates the distribution, location, and classification of lateral rib fractures using the AO/OTA classification, focusing on fracture patterns and the relationship to neighbored ribs.

**Methods:**

The study retrospectively analyzed 116 patients with 617 isolated rib fractures treated at a Level 1 trauma center over seven years. Using CT scans, fractures between the tubercle and osteochondral junction of the rib shaft were examined. Fracture type, dislocation, and location were categorized according to AO standards. The 116 patients underwent detailed statistical analysis to identify distribution patterns and correlations between fracture characteristics.

**Results:**

The fractures predominantly occurred between the fifth and seventh ribs, with a focus in the anterolateral to lateral region (40°–69°). Type A fractures were more anteriorly located, while type B fractures and dislocations shifted posteriorly. A regression analysis confirmed the significance of fracture type and dislocation in determining fracture position. Moreover, fractures showed clustering patterns, with adjacent ribs more likely to be injured. A caudal shift in fracture density and localization from the cranial to the caudal thorax was also observed.

**Discussion and conclusion:**

The findings validate the AO/OTA classification for rib fractures, highlighting the need for refined subsegmental divisions within the rib shaft for more precise clinical application. The study underscores the relationship between fracture location, type, and associated injuries, advocating for multicenter studies and a comprehensive classification system for thoracic trauma. This could enhance our understanding of injury patterns and inform treatment strategies.

## Introduction

The relevance of rib fractures and rib series fractures is becoming more and more important in everyday clinical practice, only as thoracic monotrauma, but also in the context of polytrauma [1]. For example, up to 48.2% of severely injured patients admitted to a trauma center have an injury to the bony chest wall with rib involvement. In 16.1% of patients, there is an unstable chest wall ('flail chest'), i.e. an instability of the thoracic wall due to rib series fractures with multi-fragment fractures [2].

There are different concepts for the treatment of fractures of the bony chest wall. In the case of uncomplicated, stable rib fractures, conservative treatment can often be effective. Under certain conditions, however, surgery is necessary. Possible indications for this include respiratory insufficiency, delayed fracture healing or pseudarthrosis, an unstable chest wall, severe therapy-resistant pain and complicated for example overlapping or impressed fractures [3–7].

The frequency of concomitant injuries also varies depending on the severity of the injury, but of course it is one of the decisive factors for therapy strategy and outcome. Schulz-Drost et al. showed that organ-related chest injuries occurred less frequently in polytraumatized patients with rib series fractures than in flail chests. 46% of patients with rib fracture had a pneumothorax, 54% had a flail chest, 22.4 versus 34.94% had a hemothorax, 40.9 versus 46.0% had a pulmonary contusion [2].

From these register data, a correlation of injury severity of the ribs and frequency of additional injuries can be derived. However, it is difficult to prove a connection between morbidity and mortality with an exact distribution pattern of the rib fractures, as there is simply no regular recording of the location and morphology of the rib fractures.

Even in the AO/OTA classification system of human bones, which has been tried and tested for decades, the thoracic wall was only included in the last revision in 2018 [8].

**Fig. 1:**
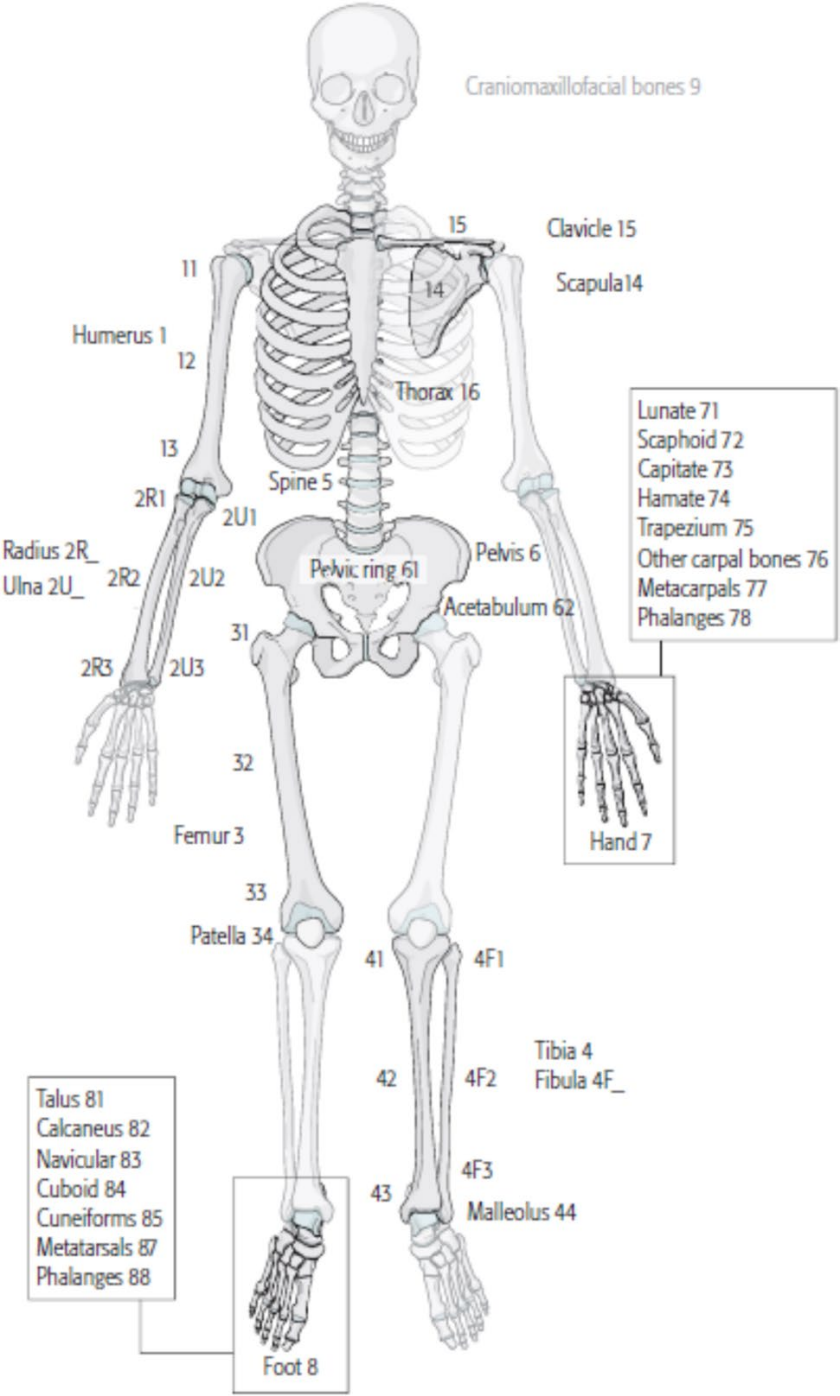
AO/OTA classification—body regions (AO Foundation, Orthopaedic Trauma Association 2018)

**Fig. 2:**
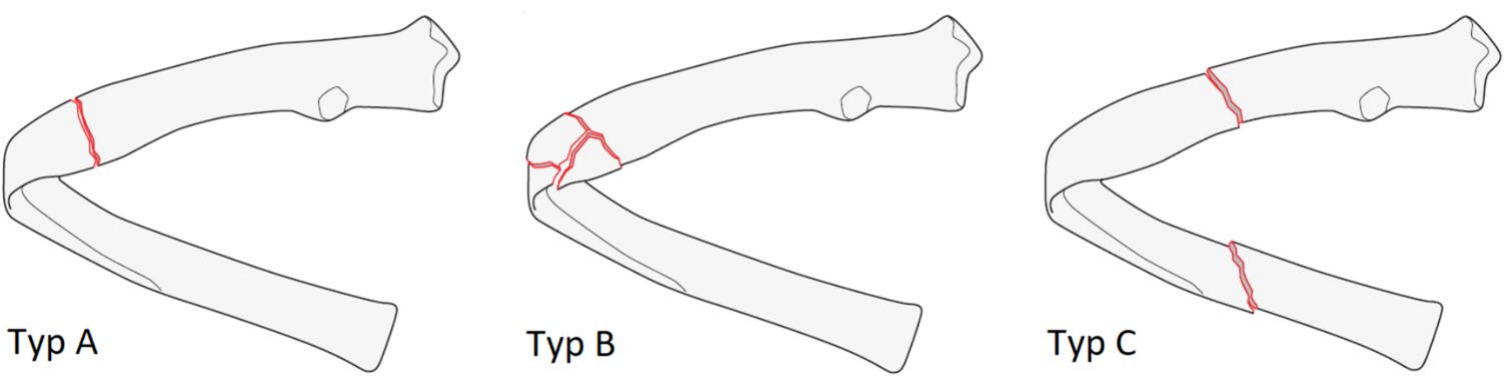
AO/OTA classification—fracture types of the ribbed shaft (AO Foundation, Orthopaedic Trauma Association 2018)

The thoracic wall was defined as region 16 with 12 pairs of ribs and the sternum (Fig. 1). Each rib was defined analogously to the long bones of humans with a central shaft segment as well as proximal (posterior—costotransverse rib portion) and distal (anterior—cartilage portion) end segment. The shaft segment is the longest part of the ribs, and its fractures are familiarly divided into A (simple fracture), B (locally multi-part fracture) and C fractures (segment fracture) (Fig. 2).

The latest revision of the AO/OTA classification therefore describes each rib with a number and the morphology of rib fractures in the respective subsegment for the first time, so that these can be described in a reproducible manner. This could enable correlations between injury patterns and clinical outcome in the future [9].

The present study will primarily investigate which ribs break how often, at which location and with which fracture type on the rib shaft.

Secondarily, it will investigate whether there are clusters of fracture locations and correlations with neighboring ribs.

The AO/OTA classification system currently provides a reproducible framework for the description and classification of single rib fractures but lacks a refined subsegmental subdivision of the rib shaft. This study aims to fill this gap by examining fracture patterns and their relationship to adjacent ribs, which could optimize the diagnosis of multi-segmental rib fractures as well as serial rib fractures, improve treatment strategies and predict outcomes.

## Materials and methods

### Selection of the patient collective

The selection of the patient collective was carried out as part of a retrospective study involving all hospitalized patients of a Level 1 trauma center with rib fractures or sternal fractures from 2010 to 2016. The patients were detected with a search for the ICD-Codes S22.31, S22.32, S22.41, S22.42, S22.43, S22.44, S22.45 (data set 1) and S22.2 (data set 2). A total of 1734 patients were first analyzed for CT scan, all patients without CT scan (914) were excluded. The remaining 820 patients and their CT scans were also checked for suitability for the study. A further 126 patients were excluded if they had received a CT scan, but this was either not temporally related to the fracture event, had inadequate image quality (e.g. insufficient slice thickness, only rudimentary imaging of the thoracic wall, etc.) or had iatrogenic fractures caused by surgery (e.g. osteotomies) on the thorax or already consolidated fractures. This left 694 patients for further analysis. After removal of duplicates, a collective of 632 patients remained to be analyzed. The remaining 632 patients were divided into three sub-collectives (anterior sub-collective (at least one fracture of the anterior chest wall (sternum and rib cartilage)), a lateral sub-collective (isolated fractures of the lateral wall and posterior sub-collective (at least one fracture of posterior wall (medial to the tuberculum of the rib and at the costovertebral joint joint) if there were no anterior fractures) by analyzing the CT-findings and screening the CT images. The sub-collective analyzed in this study contains patients who had isolated fractures of the lateral wall.

**Fig. 3:**
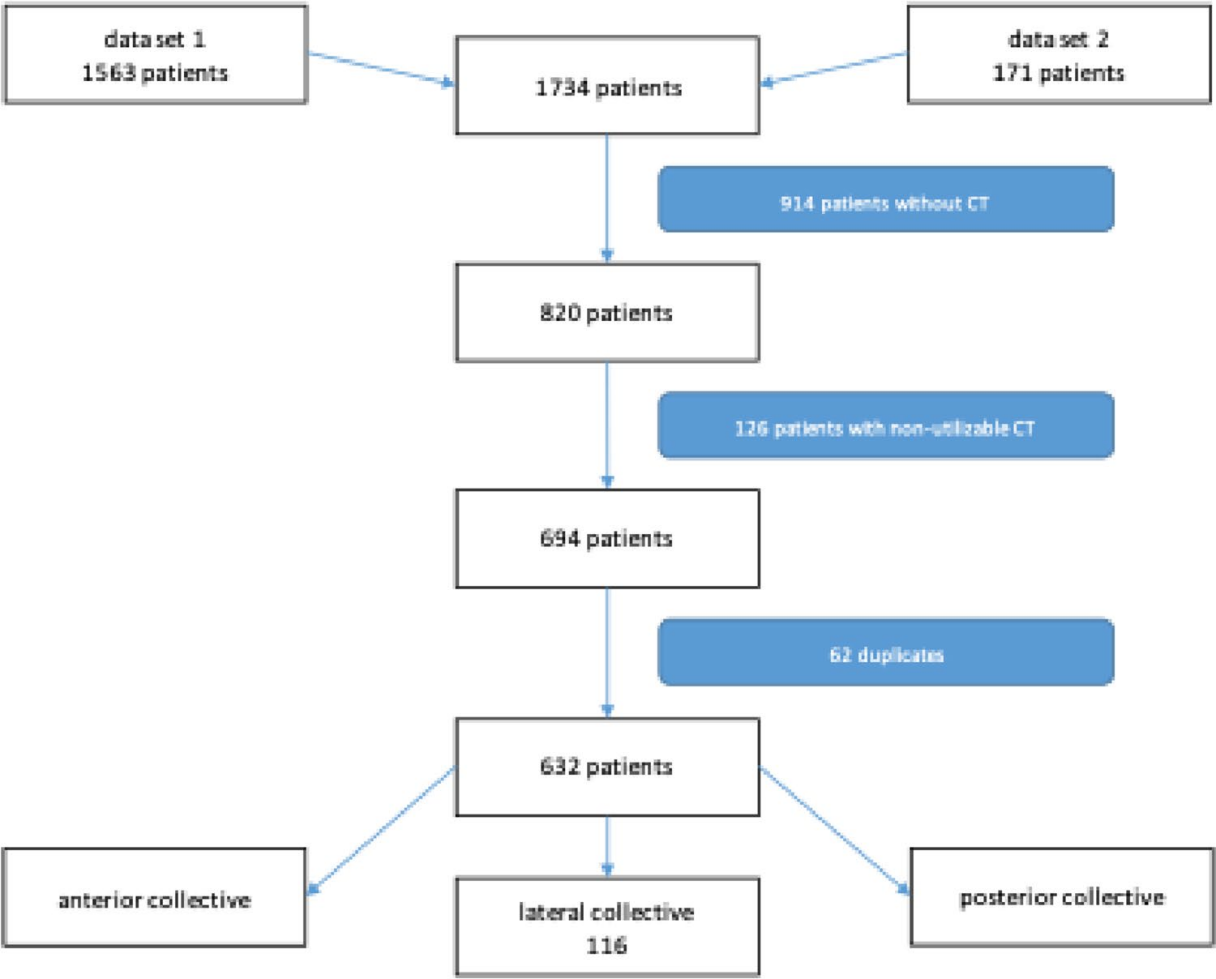
Creation of the patient collective

A collective of 116 patients (43 female, 37.1%) was formed and included in the further detailed analysis. The following flowchart provides an overview of the process of creating the collective (Fig. 3).

### Fracture analysis

All CT datasets were examined for the presence of rib shaft fractures in three dimensions– axial, coronary and sagittal.

Both the number of fractures per case and per rib as well as their morphology were examined and documented. Each fracture was classified according to the current proposal of the AO /OTA for the bony thorax. A distinction is made between an A fracture (simple fracture) and a B fracture (local multi-fragment fracture, within a rib width or wedge fracture). If several fractures occur within the rib shaft, i.e. between the osteochondral junction and the tubercle of the rib, they are classified as C fractures. C fractures are segment fractures that result from single type A or B fractures.

In the context of this study, a dislocation was defined as a displacement of the fragments of the fracture by at least one cortical width from each other.

### Method of measurements

**Fig. 4:**
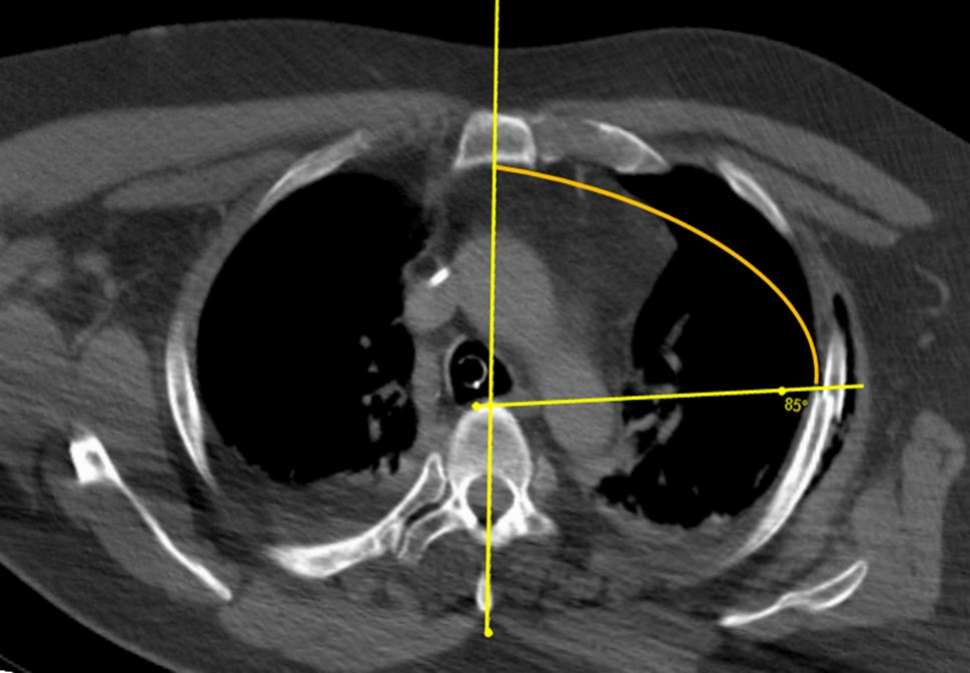
Example of measuring the position of fractures in angular degrees to the center of the body

The position of each fracture was determined in the axial CT image using the DICOM- Viewer Synedra View (synedra information technologies GmbH, Innsbruck) and measured in angular degrees in relation to the sagittal center of the body (Fig. 4). The anterior edge of the vertebra was defined as the reference point, analogous to Chest Deformity Scores such as the Haller Score. Its position is independent of congenital and trauma-associated deformities of the thoracic wall.

**Fig. 5:**
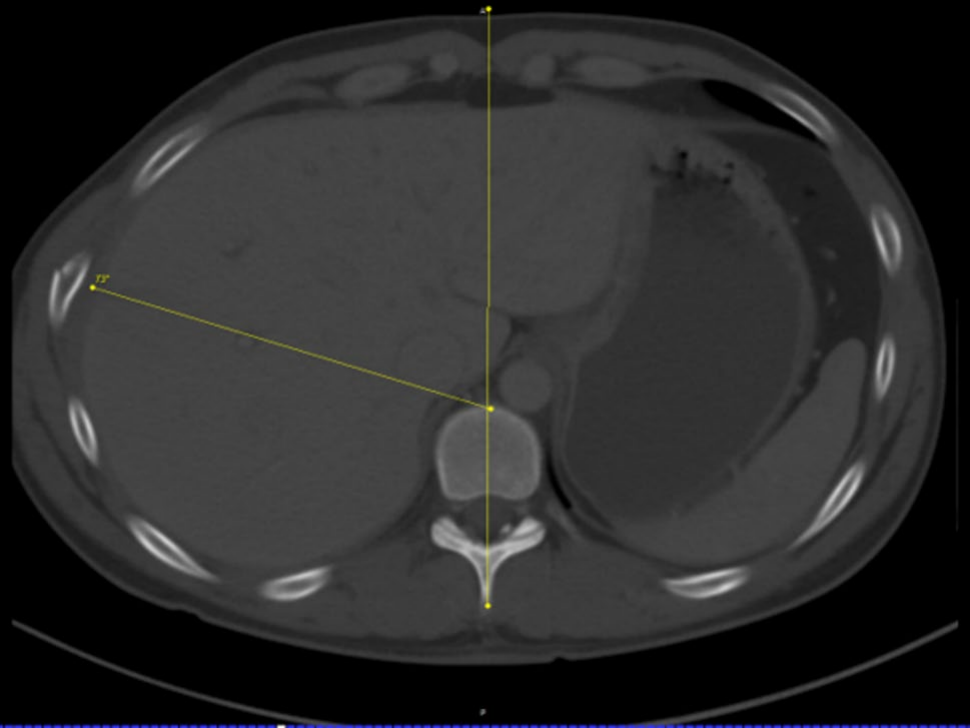
Measuring at the linea alba

Thus, the apex of the angle was centered on the anterior edge of the respective vertebral body, while the fixed limb was located on a line from the processus spinosus, center of the vertebral body and center of the sternum or further caudally in the linea alba of the abdominal wall (Fig. 5). In simple fractures, the tip of the variable limb was placed directly on the fracture edge, whereas in complicated fractures the distal end of the posterior fracture fragment served as a reference and measuring point. For fractures that were more than one rib width apart, each fracture was recorded and measured as a single fracture.

### Statistics

A regression analysis was performed to investigate the fracture position in relation to the fracture type and the dislocation.

In addition, it was examined whether neighboring ribs of a fracture region were more likely to suffer a fracture. The chi-square test was used for this purpose. Cramer's V was used to assess the strength of the statistical association between two or more nominal variables. A value of 0 indicates no statistical association, while a value of 1 represents a perfect association.

The results were statistically calculated and verified using the Statistical Package for the Social Sciences (SPSS, Version 21, IBM Inc., Armonk, NY, USA).

The classification was conducted by two experienced trauma surgeons, and interrater variability was assessed with a Cohen's kappa of 1.

## Results

### Collective

The average age at admission to hospital was 57 years (± 19.7). The average length of stay in hospital was 19.4 days (± 16.6) with a maximum of 92 days.

### Fractures in general

A total of 617 rib fractures have been detected in the lateral sector between the osteochondral junction and the tubercle of the rib. This corresponds to an average of 5.3 fractures and a median of 4.5 fractures per patient. The 617 fractures are divided into 528 type A fractures (85.6%) and 89 type B fractures (14.4%). Type C fractures, which are composed of at least two of these fractures, occur 79 times (12.8%). A dislocation of at least one cortical width was detected in 326 of the 617 fractures (52.8%).

**Fig. 6:**
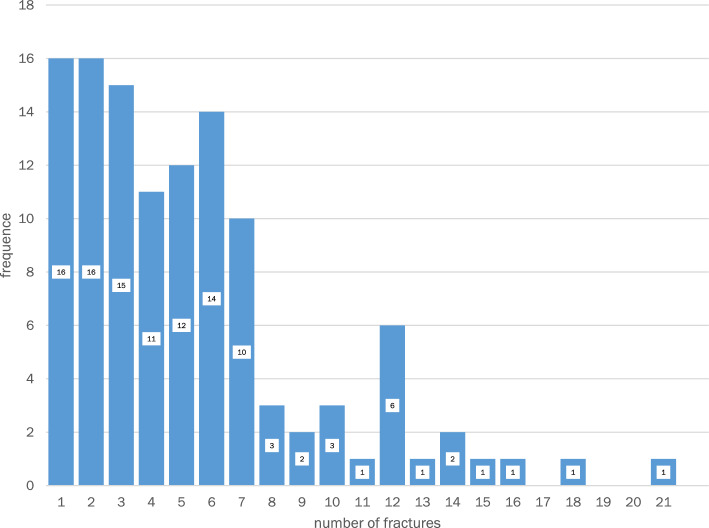
Total number of fractures per patient

In Fig. 6 the number of rib fractures per patient can be seen. Patients with one or two rib fractures occur most frequently, but the frequency of up to seven rib fractures per patient remains relatively evenly at a high level and only drops significantly from eight fractures per patient. The maximum was 21 fractures (n = 1).

The chi-square test can be used to determine the likelihood of fractures occurring in neighboring ribs. If a rib is fractured, the directly surrounding ribs are also more likely to be fractured. The observed effect decreases with each distant rib and thus clearly shows a correlation between the occurrence of adjacent or combined rib fractures. For rib three used as an example, this can be demonstrated by means of chi-square test statistics at a 99.9% significance level for the surrounding ribs two (Cramer's V = 0.648) and four and at a 99.5% significance level for ribs one (Cramer's V = 0.260) and five (Cramer's V = 0.375). Only from the sixth rib onwards is this correlation no longer significantly detectable. The same correlations exist for the other ribs.

To analyze the correlations between the number of fractures per rib over the entire thorax, the corresponding correlations were calculated. The number of fractures per rib was correlated with the number of fractures in each other rib. The following correlations were found: for the first rib, there is a weak to medium positive correlation, which is significant at least at the 95% significance level for ribs two to four. If the number of fractures of the first rib increases, then the number of fractures of the second to fourth ribs also increases. Consequently, combination injuries of these ribs in the context of one or multiple rib series fractures can be assumed.

Similar correlations were found for the following ribs: mean positive significant correlations at a significance level of at least 95% were found for the surrounding two (twelfth rib), three (first and eleventh ribs), four (seventh, ninth and tenth ribs), five (second, third, fourth and eighth ribs) and six ribs (fifth and sixth ribs). There were no significant correlations for the more distant ribs. Fractures in the cranial thorax are less likely to be combined with fractures in the caudal thorax and vice versa.

### Localization of fractures

The 617 fractures recorded ranged from 24°, as the most anterior fracture, to 140°, as the most posterior fracture. The mean value of the fracture positions is 80° (± 28.2).

A regression with fracture position as the dependent variable and fracture type and dislocation as independent variables showed a significant correlation for fracture type at a 99% level of significance and for dislocation at a 95% level of significance. The relationship between the dependent and independent variables can be described using the regression equation:

$${\widehat{Y}}_{fracture position}= {\widehat{\beta }}_{0}+{\widehat{\beta }}_{fracture type}*{X}_{fracture type}+{\widehat{\beta }}_{dislocation}*{X}_{dislocation}$$

After estimating the parameters, the following equation is obtained:

$${\widehat{Y}}_{fracture position}=\text{ 75,363}+\text{ 9,852}*{X}_{fracture type}+\mathrm{6,081}*{X}_{dislocation}$$

If fracture type A and no dislocation is present, the fracture position is 75.363° on average and under otherwise identical conditions. If a fracture type B is present, the fracture position increases by 9.852° on average and under otherwise identical conditions to 85.215°, thus shifting further posteriorly. If, on the other hand, there is a dislocation, the position increases by 6.081° on average and under otherwise identical conditions. These effects occur in combination in dislocated B fractures.

**Fig. 7:**
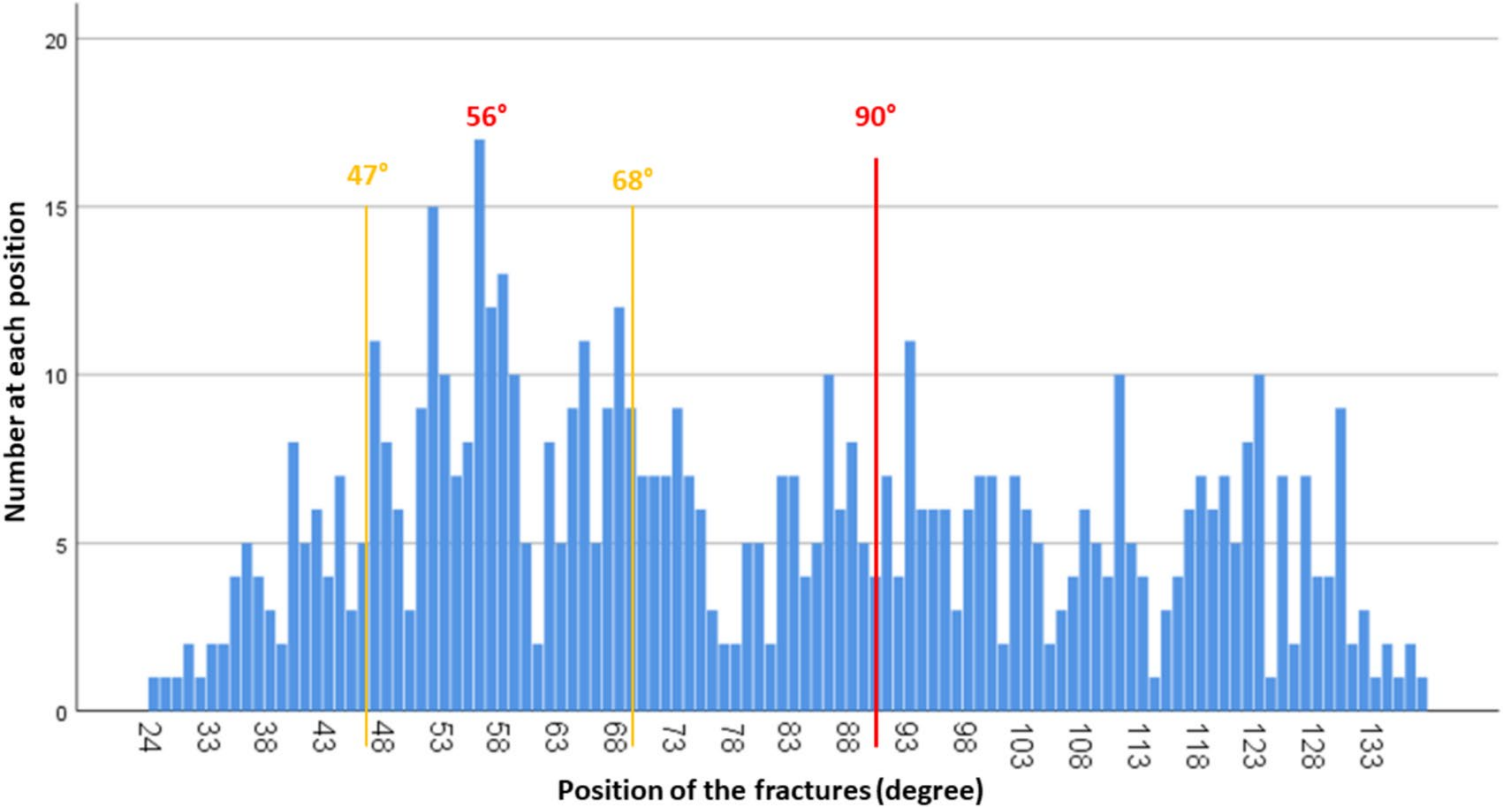
Fracture positions: total distribution of 617 fractures in 116 patients. The 90° mark shows the most lateral point of the ribs. Fractures between 47° and 68° were particularly common, with a maximum of 56°

The distribution of fractures is shown in Fig. 7. It can be seen here that fractures in the anterior shaft area (up to 40°) and posterior shaft area (from 130°) are significantly less frequent. At the 56° position, the maximum number of fractures at one degree is 17. There are only nine positions with more than ten fractures, eight of which are within a width of 22° (between 47° and 68°) in the anterolateral to lateral region of the thorax.

### Heat zone diagram

**Fig. 8:**
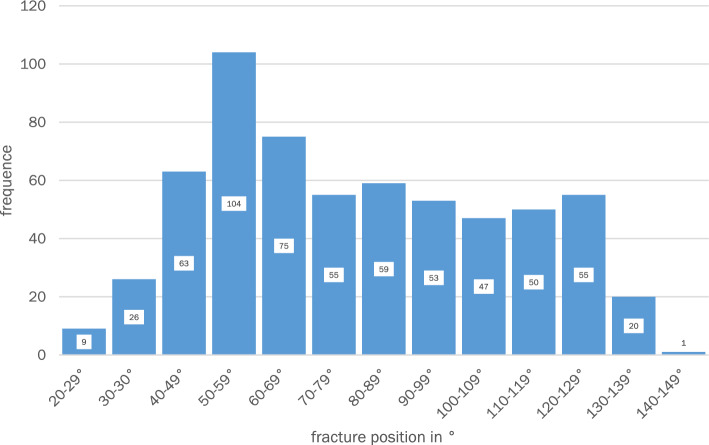
Fracture positions in 10° sectors

To better represent the distribution of fractures, the thorax was divided into 13 equal sectors. Sectors in 10° increments are used here. Due to the small-scale division into sectors with a width of 10° each, this classification is well suited to look at the distribution of fractures in smaller sectors and to identify differences. This is illustrated in Fig. 8.

There is a clear fracture focus between 40° and 69°. A total of 242 fractures (39.2%) in three of 13 sectors (23.1% of all sectors) are located within this 30° wide sector. From this it can be concluded that the mean value of 80° (± 28.2) alone is not very meaningful.

**Fig. 9:**
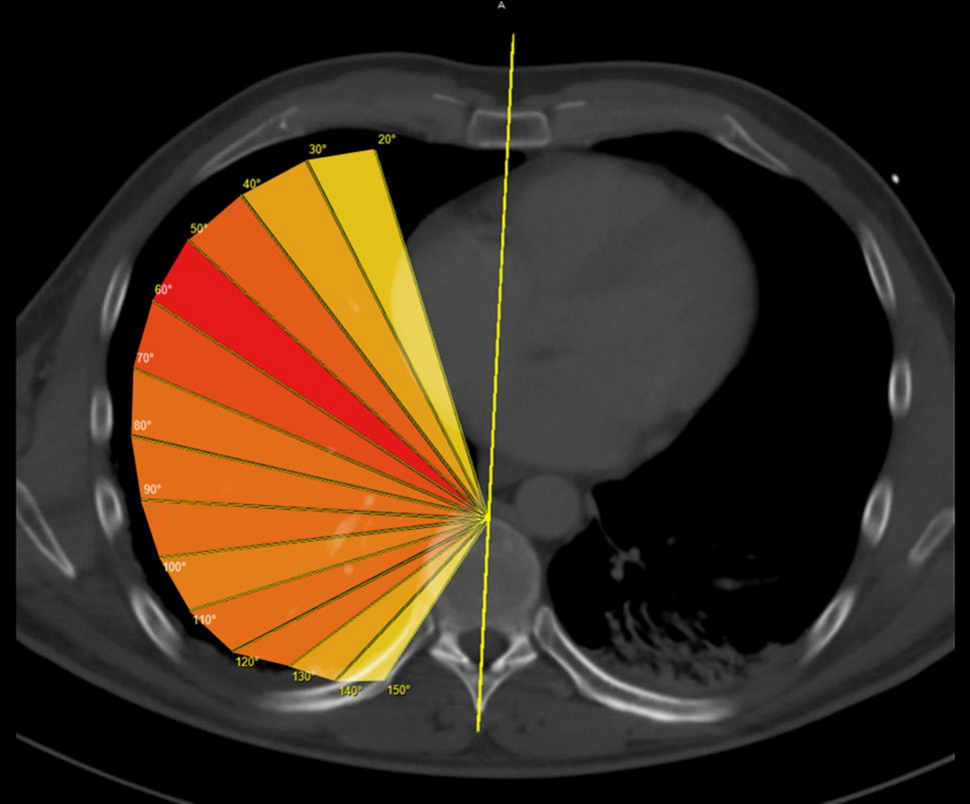
Heat zone diagram of fracture distribution

These results are shown again in Fig. 9 in the form of a heatmap. For this purpose, the 10° wide sectors on a CT image were colored differently at the level of the sixth rib. According to the fracture frequency per sector, colors ranging from yellow (few fractures) to orange to red (many fractures) were used. This representation of the fracture frequency in combination with Fig. 8 is intended to visualize the results in this area.

This figure also shows the fracture center between 40° and 69° with the core sector at 50° to 59°. However, the coloring also shows that the number of fractures occurring posteriorly remains relatively constant up to the sector from 120° to 129°. Two anterior sectors (20°–39°) and the two posterior sectors (130°–149°) stand out clearly in the thermal zone diagram due to a lower fracture frequency.

### Fracture distribution by individual ribs

**Fig. 10:**
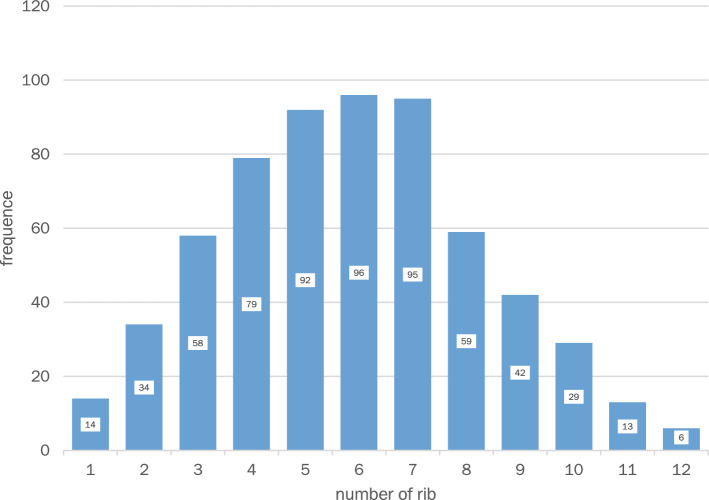
Fracture frequency per rib

The distribution of fractures and fracture positions in relation to the twelve individual ribs is shown in Fig. 10 and reveals an increasing frequency of fractures from cranial to caudal up to the sixth rib with a marked decrease from the eighth rib. The maximum is in the region of the fifth to seventh rib. Thus, 283 (45.9%) of the total of 617 fractures occur at these three ribs, although these only account for 25% of the total of twelve ribs. It can also be seen that the caudal region from rib nine onwards is rather rarely affected by fractures, accounting for 14.6% of fractures and 33.3% of the possible distribution. In the cranial region, rib fractures occur more frequently, although the first rib also has very few fractures (only 2.3%).

**Fig. 11:**
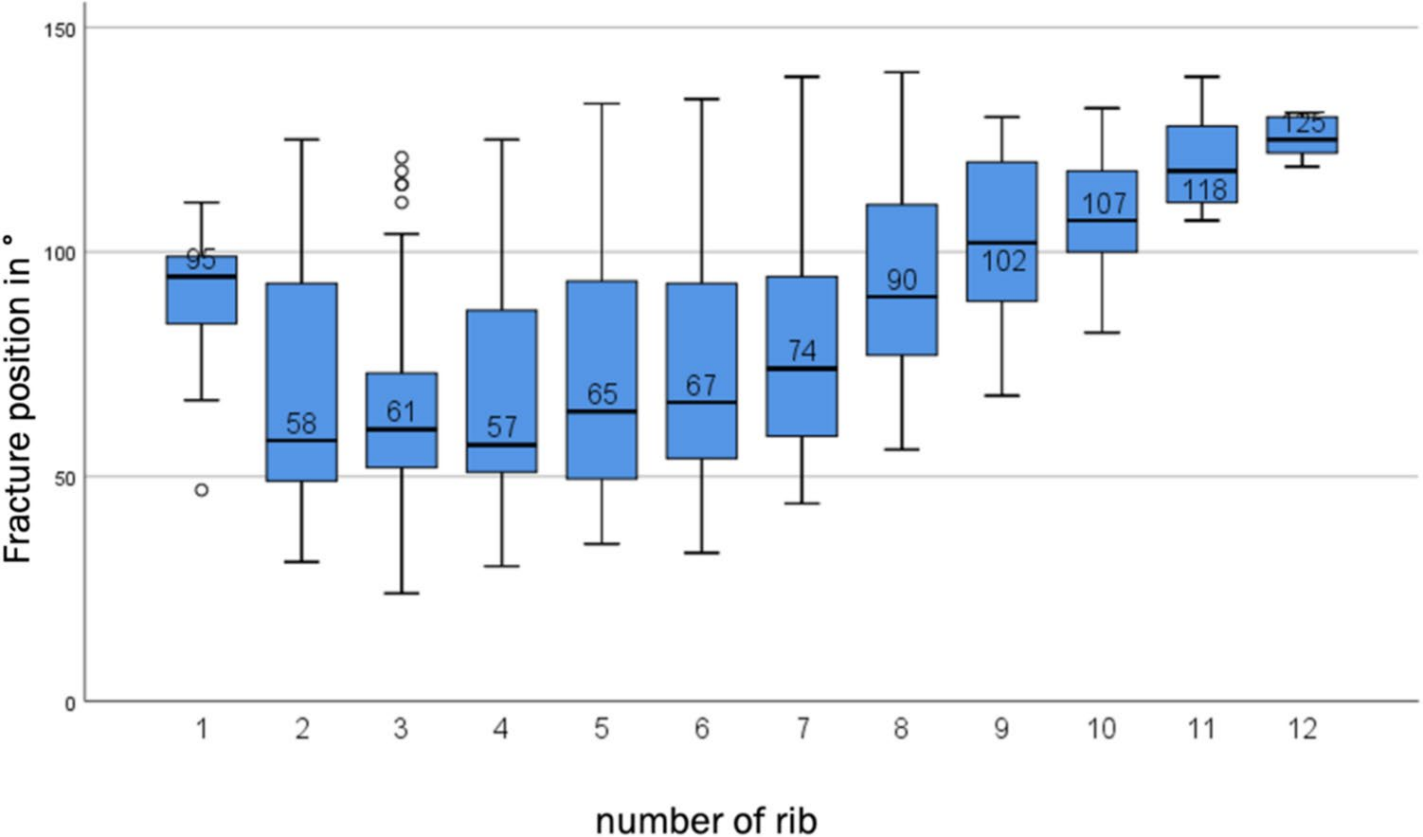
Fracture positions per rib boxplot

When analyzing the fracture localization for each individual rib, Fig. 11 shows a striking picture. The distribution of fractures shifts noticeably posteriorly from the eighth rib onwards. The fracture area is also significantly smaller, particularly in the ninth to twelfth ribs.

**Fig. 12:**
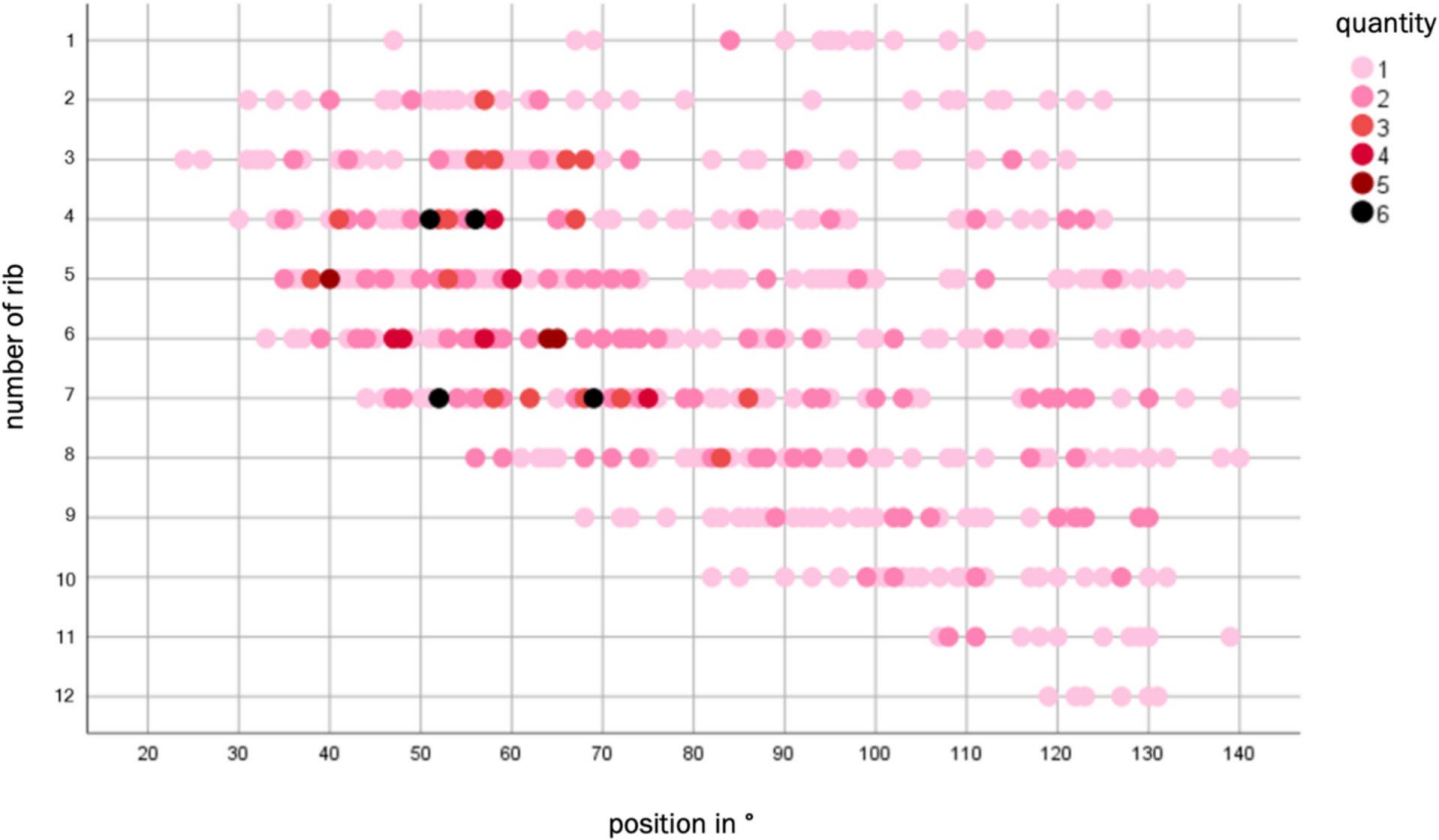
Positions of rib fractures in the thorax

To visualize the distribution of rib fractures in the entire thorax, a three-dimensional coordinate system was created from the data, which depicts the positions of the rib fractures in the thorax. This is shown in Fig. 12.

In this coordinate system, the ribs are shown from cranial to caudal on the y-axis and the positions of the fractures from anterior (20°) to posterior (140°) on the x-axis. The color of the dots reflects the number of fractures found at a point and thus, in combination with the clustering of the dots, indicates the fracture foci.

The three-dimensional coordinate system clearly depicts the distribution of fractures in the thorax and shows the shift in both the fracture centers and the range of fractures that occurred from more anterolateral in the cranial thorax to the posterolateral area in the caudal thorax.

As already shown in Fig. 10, this graph also illustrates that ribs one, eleven and twelve are fractured significantly less frequently than the other ribs, which can be explained by their anatomical position and configuration, among other things. However, this clearly distinguishes them from the other ribs.

In addition, both a fracture focus in ribs four to seven, analogous to Fig. 10, and an accumulation of fractures in the anterolateral to lateral region (40°–69°), analogous to Fig. 8, can be identified in this graph.

## Discussion

The demographic structure of the present collective correlates with the current literature. Compared to the overall collective, this sub-collective is proportionally small, as it focuses only on isolated lateral rib fractures. Patients often have additional fractures in the anterior and posterior sectors, which were not observed in this sub-collective [10, 11]. Isolated fractures of the lateral thorax segment are rare [12].

It has been shown that the general classification of the AO/OTA classification into A, B and C fractures in fractures of the rib shaft is conclusive and correlates with the clinical occurrence of these fractures. In a further study by our working group, we were able to show that this does not apply, for example, in the area of the central cartilaginous part of the ribs [11].

### Fracture localization in the vertical plane

In this study, rib fractures affecting the rib shaft in isolation are considered. On average, there were more than 5 fractures per case, in most cases one to seven fractures. At 59% of the total proportion, these were mainly distributed over the 4th to 7th ribs and there in the anterolateral region around the 55th degree of angle (Figs. 5, 6, 7, 8, 9, 10). In particular, the caudal region from the ninth rib onwards is rarely affected with only 14.6% of fractures, which is mainly due to the posterior displacement of the osteochondral junction there.

Liebsch et al. also found a similar distribution of fractures [13]. With an increase in rib fractures from ribs one to five and a decrease from rib six caudally, the authors described a bell-shaped fracture distribution in the area of ribs one to ten and an accumulation of more than 50% of the documented rib fractures in ribs four to seven, which is consistent with the results of this study. Thus, this fracture distribution in the vertical plane can be repeatedly demonstrated due to anatomical conditions and various accident mechanisms, as comparable results and fracture distributions can also be found in other studies [3, 14].

The relevance of the affected ribs is particularly evident in the associated concomitant injuries, which are associated with fractures located at different distances cranially or caudally. Due to the different categorization of ribs in the literature, the available data shows different results. For example fractures of ribs one and two are generally associated with a high absorbed kinetic energy, which points to a corresponding accident mechanism and could be an indicator of pronounced mediastinal and vascular injuries [15]. According to another study, the presence of a fracture of rib one is already a marker for life-threatening intrathoracic injuries [16]. An injury to ribs one to four is highly predictive of concomitant sternal and scapular injuries, while fractures of ribs nine to twelve are more likely to be associated with injury to solid organs in the upper abdomen [17]. In the case of fractures of the caudal ribs, the presence of injury to the liver and spleen in particular must be ruled out due to the spatial proximity [15]. However, a generally significant effect of the localization of the fractures in the vertical plane on the mortality of the patients was not found [18].

### Fracture localization in the axial plane

The localization of the rib fractures in the lateral shaft segment in the axial plane shows a fracture focus in the anterolateral to lateral region with a clear focus between 40° and 69° according to the data of this study. As can be seen in Fig. 7, the fracture maximum within this fracture focus is 56°. In the posterolateral region, on the other hand, there are only individual fracture clusters without a relevant center of gravity.

The comparability of these results is limited due to the different classification and measurement methods for rib fractures in the literature. In particular, the definition of the anterolateral, lateral and posterolateral regions is not uniformly agreed upon. Nevertheless, an accumulation of rib fractures in the anterolateral to lateral region of the thorax has been described several times. In the literature an accumulation of fractures in the lateral sections of ribs three to twelve is discribed [15]. Another study found a majority of fractures in the anterior to anterolateral region ventral to the 90° position [14]. In contrast, other authors described an accumulation of fractures in the posterolateral region [3].

This distribution is particularly important as lateral rib fractures have a significant effect on mortality, a 1.13-fold increase in mortality is described in the literature [18]. In the anterior and lateral region, the fractures are also said to have a greater influence on respiratory mechanics, as the posterior section is stabilized by the paravertebral muscles [19].

The measurement method established here enables a clear spatial positional assignment of each rib fracture regardless of any deformity of the thorax. With a little technical skill, the angular degrees could be integrated into a mapping of rib fractures, for example the unfolded rib representation [20]. From this in turn, conclusions could then be drawn about the fracture distribution in connection with accident mechanisms and presented graphically in a plausible manner. By examining further and larger collectives in a uniform approach, it would then be easier to define reliable and clinically relevant sector boundaries.

### Fracture localization of the subgroups

With regard to the subgroups, the distribution of fractures is as follows. The simple type A fractures follow the overall distribution described above, while the complex type B multi-fragment fractures show an opposite distribution with a focus in the lateral to posterolateral region between 100° and 129°.

The displaced fractures are distributed relatively homogeneously over the entire fracture range from 40° to 129°, while the non-displaced fractures have their center of gravity between 40° and 69° in accordance with the overall distribution.

Complex type B fractures, which are also displaced, occur primarily in the posterolateral region of the ribs. Although the classification of fracture types is not congruent, the literature also indicates that complicated multi-fragment fractures are mainly found in the posterolateral to posterior region of the ribs [13]. In addition, the anterolateral and lateral focus of non-displaced fractures documented in this study has also already been described by the authors.

### Shift of the fracture centers

The three-dimensional coordinate system in Fig. 11 clearly shows the shift in fracture density in the bony thorax for this collective from more anterior in the cranial thorax (ribs one to three) via lateral in the middle thorax (ribs four to eight) to posterior in the caudal thorax (ribs nine to twelve). This is only partly due to the osteochondral junction, which shifts posteriorly in the craniocaudal direction, limiting the possible fracture area of the lateral shaft segment. This is illustrated in particular by the fracture distribution of ribs eleven and twelve, on which all fractures are located far posteriorly. Irrespective of this, the trend towards a shift in fracture density is also clearly recognizable.

It can therefore be stated that a clear fracture center can be seen in the middle area of the thorax from ribs four to seven and that the fracture density shifts posteriorly in a craniocaudal direction over the entire thorax. Similar courses and clusters of rib fractures can be found in the literature [13].

### Relationships between fractures

In addition to the pure localization and distribution of fractures, the correlations between the fractures are also important. There is a clear correlation here in the occurrence of adjacent or combined rib fractures. It was also found that fractures in the cranial thorax are less likely to occur together with fractures in the caudal thorax and vice versa.

Correlations of this kind are relevant. According to several studies, the probability of combined injuries, particularly around the chest wall and the lungs, and the mortality rate increase with an increasing number of rib fractures. There is also a direct correlation between the increase in the number of rib fractures and pulmonary injuries and secondary diseases, such as pneumonia [17, 21].

It is also evident that a number of three or more rib fractures, especially in patients over 65 years of age, is a risk factor for increased mortality [22].

The correlation between rib fractures appears understandable based on the most common accident mechanisms, as thoracic trauma in Germany occurs in particular due to blunt trauma mechanisms in traffic accidents or falls from a great height, which usually result in local damage to the chest wall [9].

## Conclusions

The investigation of the localization of rib fractures revealed significant results in the axial and vertical planes, which are related to the fracture types and the dislocation of the fractures. The occurrence of fracture foci and their displacement across the thorax also underline the relevance of these parameters. As part of the further development of the AO/OTA classification, these aspects should also be considered in future studies on concomitant injuries as well as the morbidity and mortality of thoracic trauma.

This directly leads to a key insight of our study: the necessity of understanding fracture systems rather than focusing solely on rare isolated fractures. Our analysis also highlights the frequent involvement of adjacent ribs, emphasizing the importance of a systematic approach. We want to clarify that our study does not aim to introduce new classifications but rather to provide foundational data to enable a better description of combined injuries. The careful examination of a refined cohort, even if it represents only a small portion of the overall picture, offers valuable and high-quality insights.

In principle, the AO/OTA classification can be applied to the classification and description of individual fractures. For the future, however, the present study suggests that it is necessary to divide the rib shaft segment into further subsegments with reproducible boundaries. The 10° sectors shown would be difficult to apply, whereas a subdivision into anterolateral and posterolateral with the dividing point at 90° would be easy to apply. A three-way division of the socket into anterolateral, lateral and posterolateral subsegments is also conceivable and has already been proposed by the CWIS [23]. It is also used in CIID, but it is difficult to determine the sector boundaries.

For further research, the introduction of an AO/OTA classification for the bony thorax requires a register with image data sets that is as multicenter as possible. The clinical courses must then be correlated with the fracture morphology to examine correlations. Further investigation of fracture types, dislocations and fracture modifiers would also appear to be useful for this purpose.

However, the greatest challenge will be to transfer multiple individual fractures into a classification system that can describe and compare summarized injury patterns including all ribs, the sternum and their regions on the thoracic wall. This is exactly what we need to be able to evaluate clinical courses and treatment strategies.

### Limitations

This study, however, has several limitations that should be considered when interpreting the findings. First, this is a purely morphological study aimed at identifying common fracture patterns in the lateral rib shaft. While the findings highlight the utility of the AO/OTA classification, clinical outcomes and treatment strategies were not directly assessed. Additionally, the study focused solely on lateral rib shaft fractures, excluding those medial to the rib angle or associated with the sternum or spine, which limits the generalizability of the findings to all types of rib fractures and may not reflect real-world polytrauma scenarios.

Although the AO/OTA classification system is reproducible and provides a solid framework by dividing fractures into types A, B, and C, these categories alone may not fully inform treatment decisions or predict outcomes. Further refinement of the system to include subsegmental divisions is required and will need additional validation.

Moreover, the data were collected from a single Level 1 trauma center. Multi-center studies are essential to validate these findings across diverse populations. Another limitation is that the study did not distinguish between left, right, or bilateral rib fractures, though these factors may influence clinical outcomes. Future research should address this aspect to further enhance the clinical relevance of rib fracture classification.

By addressing these limitations, future studies can provide more comprehensive insights into rib fracture systems, refine classification methods, and improve their role in guiding treatment strategies and predicting outcomes.

## Data Availability

The data that support the findings of this study are available from the corresponding author upon reasonable request.
